# The endogenous anti-angiogenic VEGF isoform, VEGF_165_b inhibits human tumour growth in mice

**DOI:** 10.1038/sj.bjc.6604309

**Published:** 2008-03-18

**Authors:** E S Rennel, E Waine, H Guan, Y Schüler, W Leenders, J Woolard, M Sugiono, D Gillatt, E S Kleinerman, D O Bates, S J Harper

**Affiliations:** 1 Microvascular Research Laboratories, Department of Physiology and Pharmacology, School of Veterinary Science, University of Bristol, Southwell Street, Bristol BS2 8EJ, UK; 2 Division of Pediatrics Research, Unit 87, University of Texas MD Anderson Cancer Center, 1515 Holcombe Blvd., Houston, TX, USA; 3 Radboud University Nijmegen Medical Centre, Department of Pathology, Nijmegen, The Netherlands; 4 Bristol Urological Institute, Southmead Hospital, Westbury-on-Trym, Bristol, UK

**Keywords:** VEGF, angiogenesis, anti-angiogenesis, splice variant, cancer growth, *in vivo* tumour inhibition

## Abstract

Vascular endothelial growth factor-A is widely regarded as the principal stimulator of angiogenesis required for tumour growth. VEGF is generated as multiple isoforms of two families, the pro-angiogenic family generated by proximal splice site selection in the terminal exon, termed VEGF_xxx_, and the anti-angiogenic family formed by distal splice site selection in the terminal exon, termed VEGF_xxx_b, where xxx is the amino acid number. The most studied isoforms, VEGF_165_ and VEGF_165_b have been shown to be present in tumour and normal tissues respectively. VEGF_165_b has been shown to inhibit VEGF- and hypoxia-induced angiogenesis, and VEGF-induced cell migration and proliferation *in vitro*. Here we show that overexpression of VEGF_165_b by tumour cells inhibits the growth of prostate carcinoma, Ewing's sarcoma and renal cell carcinoma in xenografted mouse tumour models. Moreover, VEGF_165_b overexpression inhibited tumour cell-mediated migration and proliferation of endothelial cells. These data show that overexpression of VEGF_165_b can inhibit growth of multiple tumour types *in vivo* indicating that VEGF_165_b has potential as an anti-angiogenic, anti-tumour strategy in a number of different tumour types, either by control of VEGF_165_b expression by regulation of splicing, overexpression of VEGF_165_b, or therapeutic delivery of VEGF_165_b to tumours.

Vascular endothelial growth factor (VEGF-A) is the principal angiogenic promoter in most, if not all, cancers acting primarily on endothelial cells through its cognate receptors VEGF-R1 and VEGF-R2. VEGF is upregulated by hypoxia (Shweiki *et al*, 1992), and by overexpression of oncogenes such as mutant ras (Rak *et al*, 1995) and c-myc (Mezquita *et al*, 2005) in tumours, and stimulates the migration of endothelial cells, sprouting of blood vessels, and generation of new vessels from existing vasculature in tumours (reviewed in Ferrara, 2004). The resulting sustained blood flow, oxygen supply and waste removal enables more rapid growth of the tumour. Anti-VEGF therapy is now an additional therapeutic strategy to surgery, chemotherapy and radiotherapy, and recent trials of antibodies to VEGF as adjuvant therapy have shown significant clinical benefit in colorectal cancer (Hurwitz *et al*, 2004), renal carcinoma, non small cell lung, ovarian and other cancers.

Vascular endothelial growth factor is generated as multiple isoforms by alternative splicing of mRNA from 8 exons (Houck *et al*, 1991). Conventional VEGF isoforms are pro-angiogenic, pro-permeability vasodilators. These isoforms contain exons 1–5 and 8a, with a variable contribution from exons 6a, 6b, 7a, and 7b, resulting in a family of peptides identified numerically by their amino-acid content, each with different heparin-binding properties (Houck *et al*, 1991). These are generically identified as VEGF_xxx_, where xxx refers to the number of amino acids. In 2002 (Bates *et al*, 2002) and 2004 (Woolard *et al*, 2004) we identified a sister family of isoforms of identical lengths and exon structure apart from the C-terminal exon in which distal splicing results in an alternate open-reading frame of six amino acids (exon 8b rather than 8a, SLTRKD *vs* CDKPRR) – generically referred to as VEGF_xxx_b (e.g. VEGF_165_b, VEGF_121_b (Perrin *et al*, 2005), VEGF_189_b (Miller-Kasprzak and Jagodzinski, 2008)). These isoforms are antiangiogenic and downregulated in renal tumours and metastatic melanoma (Bates *et al*, 2002; Pritchard-Jones *et al*, 2007). This antiangiogenic activity, generated by receptor binding but only weak receptor activation (Cebe Suarez *et al*, 2006) and inhibition of downstream VEGF-R2 signalling, has led to the hypothesis that VEGF_165_b, or manipulation of C-terminal VEGF splicing to enhance more distal splicing, may be useful therapeutic tools in cancer.

We have previously shown that VEGF_165_b is present in a range of normal tissues (Bates *et al*, 2002; Woolard *et al*, 2004) and there is a downregulation of the antiangiogenic VEGF_165_b protein in malignant prostate cancer (Woolard *et al*, 2004) and metastatic melanoma (Pritchard-Jones *et al*, 2007) and mRNA in renal carcinoma (Bates *et al*, 2002). We also showed that VEGF_165_ expressing tumours grew faster than VEGF_165_b expressing tumours, suggesting that VEGF_165_b was not angiogenic. Moreover, we showed that tumours in which VEGF_165_b expressing cells were mixed with VEGF_165_ expressing cells grew more slowly than cells only expressing VEGF_165_ (but more quickly than cells expressing VEGF_165_b alone). This latter finding rules out a possibility that VEGF_165_b could be angiogenic in the presence of VEGF_165_. However, neither that, nor any other study, has shown whether VEGF_165_b can inhibit tumour growth, whether it can inhibit VEGF_165_-mediated tumour growth, or whether it can do so by inhibiting angiogenesis.

In this study, we have examined the effect of VEGF_165_b overexpression on tumour growth, and VEGF_165_-mediated angiogenesis of prostate and renal cell carcinoma and on growth of Ewing's sarcoma cell and metastatic melanoma in xenografted mouse models.

## MATERIALS AND METHODS

### Human tissue and RT–PCR of human TURP chips

Frozen prostate chips were obtained from patients undergoing transurethral resection of the prostate (TURP) for lower urinary tract symptoms with benign prostatic hyperplasia and advanced prostate cancer (stage T3 Nx M0-1; UICC2002). The use of tissue was approved by the *NBHST* Ethical Committee.

Fifty to 100 mg of 26 TURP tissue (nine malignant, 17 benign prostatic hypertrophy) was homogenised in Trizol reagent (Life Technologies Inc., Rockville, MD, USA) and mRNA was extracted by using the method of (Chomczynski and Sacchi, 1987). Eight microliters of RNA were treated with RNase free DNase (Promega, Madison, WI, USA) according to the manufacturer's guidelines to prevent genomic DNA contamination and mRNA was reverse transcribed using Moloney murine leukaemia virus reverse transcriptase and poly-d(T). cDNA was then amplified using intron spanning primers that detect VEGF_165_b only, even in the presence of 1000 fold greater concentration of VEGF_165_ mRNA (Bates *et al*, 2002). The cDNA was amplified using 1 *μ*M intron-spanning primers designed to detect VEGF_xxx_b (Bates *et al*, 2002) (Exon 4, 5′-GAGATGAGCTTCCTACAGCAC-3′ and (Exon8b/7, 5′-TTAAGCTTTCAGTCTTTCCTGGTGAGAGATCTGCA-3′) or VEGF_xxx_ with exon 8a (5′-CACCGCCTCGGCTTGTCACAT-3′), together with 1.2 mM MgCl_2_, 200 *μ*M dNTPs, and 1 unit of Taq DNA polymerase (Abgene), cycled 35 times, at 94°C for 30 s, 63°C for 30 s and 72°C for 60 s. *β*2-microglobulin was used as control amplification (*β*2 forward primer 5′-GCATCATGGAGGTTTGAAGATG-3′, *β*2 reverse 5′-TAAGTTGCCAGCCCTCCTAGAG-3′) at 55°C annealing temperature leading to a 220 bp product. Full-length VEGF_165_b or VEGF_165_ in pcDNA3 vector were used as positive and negative controls. PCR products were run on 3% agarose gels. PCR bands were excised and extracted using Qiaex (Qiagen, Crawley, UK), cloned using a TOPO TA Cloning® Kit (Invitrogen, Paisley, UK) and confirmed by sequencing.

### Establishment of overexpressing tumour cells

All media and supplements were from Gibco/Invitrogen if not otherwise stated. PC3 cells were kindly donated by Professor J Masters from UCH, University of London. Ewing's sarcoma cell line, TC71, renal cell carcinoma cell line, CAKI, prostate cell line, PC3, and melanoma cell line, Mel57, were grown in Eagle's modified essential medium supplemented with 10% fetal calf serum (FCS), 2 mM L-glutamine, 1 mM sodium pyruvate, 1% non-essential amino acids, NEAA, 2% minimum essential medium vitamin solution, minimum essential media (Sigma Aldrich, Dorset, UK) supplemented with 10% FCS, 1% L-glutamine, 1% penicillin/streptomycin, 1% NEAA or McCoy's 5A medium supplemented with 10% FCS and Ham F-12 supplemented with 10% FCS, 1% penicillin/streptomycin, 1.5 g l^−1^ sodium bicarbonate (Sigma Aldrich), respectively.

For transfection, 90% confluent cells in 6-well plates were transfected with 0.5 *μ*g of plasmid DNA (pcDNA empty vector, pcDNA-VEGF_165_, pcDNA-VEGF_165_b or equal amounts of VEGF_165_ and VEGF_165_b plasmids) using Lipofectamine 2000 (Invitrogen, Paisley, UK) according to manufacturers protocols and Optimem media. Cells were selected with 375 *μ*g ml^−1^ of geneticin and maintained at 125 *μ*g ml^−1^. Transfected cells were analysed by an in-house developed VEGF_165_b-specific enzyme-linked immunosorbent assay (ELISA) (Perrin *et al*, 2005) or a commercial panVEGF ELISA (Duoset, R&D Systems), that detects all isoforms of VEGF-A, to validate expression levels in selected cells. ELISA was performed either according to manufacturers instructions or as previously described (Woolard *et al*, 2004). Conditioned media from 200 000 cells was collected and was analysed by ELISA after 48 h incubation. Mel57 cells were transfected with plasmid pIRESneo containing the VEGF_165_ or VEGF_165_b cDNA essentially as described before (Kusters *et al*, 2007).

### Migration of cells with conditioned media

Cultured supernatants from TC71, empty vector transfected TC71 or TC71/VEGF_165_b cells were collected. Transwells (Costar, Cambridge, MA, USA) were pretreated with serum-free medium at 37°C for 1 h before seeding with human dermal microvascular endothelial cells, (HMVECs)at 1 × 10^5^ per well in 100 *μ*l endothelial basal media (Cambrex, Baltimore, MD, USA) with 0.1% fetal bovine serum. These cells are commercially available cells generated from human foreskin tissue. The transwells were then inserted into 24-well plates containing 600 *μ*l of conditioned medium and incubated at 37°C for 6 h to allow HMVEC cells to migrate. Cells on the upper side of the filter were removed with cotton swabs. Migrated cells on the lower side of the filter were fixed and stained with haematoxylin and eosin. The number of migrated cells was counted under a binocular microscope.

### [^3^H]-thymidine incorporation of cells with conditioned media

Human dermal microvascular endothelial cells (3 × 10^3^) were seeded into 96-well cell culture plates and allowed to adhere for 5 h before the addition of conditioned medium from TC71 cells, empty vector transfected TC71 cells or TC71/VEGF_165_b cells. Triplicate wells were used for each group. The cultures were labelled with 0.2 *μ*Ci per well of [^3^H] thymidine (ICN Biomedicals Inc., Radiochemicals Division, Irvine, CA, USA) during the last 24 h of a 48 h incubation. At the end of incubation, the cells were washed two times with HBSS, and 0.1 ml of 0.1 N KOH was added to lyse the adherent cells. The radioactive incorporation was determined using a plate harvester (Brandal Biomedical Research and Development Lab Inc., Gaithersburg, MD, USA) and Beta Plate Counter (Wallac 1450 Micro beta Counter, Perkin Elmer Life Sciences, Turku, Finland).

### *In vitro* growth analysis of cells

For direct counting and proliferation of transfected CAKI cells, 30 000 cells per 24 well were seeded out in triplicates. Cells were maintained in media supplemented with 0.01% or 10% FCS and were counted after 24 or 48 h. The doubling time for each cell population was calculated using Prism software. The metabolic rate was analysed by seeding 5000 cells per 96 well in media supplemented with 0.01% FCS. After 24 or 48 h 50 *μ*l of metabolic reagent was added (CellTiter 96® Aqueous One solution, Promega, Madison, WI, USA). Cells were incubated for 4 h at 37°C and analysed at 490 nm.

### Animal housing and xenograft model

One million Ewing sarcoma or Mel57, 8 million CAKI, 3 million PC3 overexpressing cells were injected subcutaneously into the back of unanaesthetised nude mice in 100 *μ*l sterile PBS. Xenotransplanted tumours were measured by calliper and tumour volume was calculated according to (length × width × (length+width)/2). Mice were culled by cervical dislocation when tumours reached 16 mm in any direction and organs and tumours were removed and snap-frozen or fixed in 4% PFA followed by paraffin embedding and 5 *μ*m sections were generated and stained with CD31/PECAM. Slides were boiled for 10 min in 0.01 M sodium citrate boiling, followed by blocking in 5% goat serum for 1 h, overnight incubation with 2.5 *μ*g ml^−1^ anti-mouse PECAM antibody (Pierce Endogen) and for 1 h in 5 *μ*g ml^−1^ ALEXA Fluor 488 goat anti hamster IgG (Invitrogen, Molecular Probes). Vessels were counted in six different fields at × 20 magnification and verified at × 40 magnification. Nude mice were kept under appropriate specific pathogen-free housing facilities according to government guidelines and procedures were carried out according to national guidelines and regulations.

### Statistical analysis

Statistical analysis was performed using Prism. All data are given as mean±s.e.m. if not otherwise stated. Number of benign TUR and malignant chips expressing VEGF_165_b was compared using Fisher's exact test. For tumour growth one-way analysis of variance (ANOVA) followed by Newman–Keuls' *post hoc* test (prostate) or two-way ANOVA followed by Bonferroni *post hoc* test (renal carcinoma) were used as data allowed. Weight, blood score, proliferation, cell doubling and metabolic rate were analysed by one-way ANOVA followed by Newman–Keuls' multiple comparison *post hoc* test. *P*<0.05 was considered significant.

## RESULTS

### VEGF_165_b is downregulated in malignant prostate tumours compared to benign prostate tissue and reduced tumour growth in xenograft model

**Figure 1:**
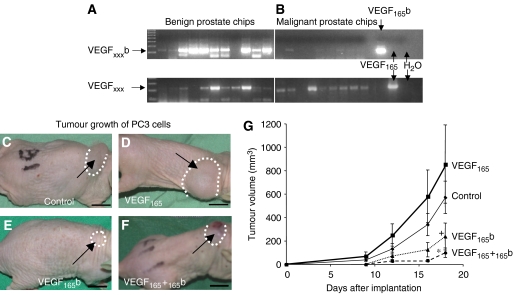
The antiangiogenic VEGF_165_b is downregulated in human malignant prostate tumour and reduces prostate tumour growth. Anonymous examples of VEGF_xxx_ and VEGF_xxx_b mRNA expression in TURP chips from benign prostate hypertrophy (**A**) and malignant prostate cancer (**B**). (**A**) RT–PCR of mRNA extracted from benign prostate chips using primers to detect VEGF_xxx_b and VEGF_xxx_. Ten out of 11 benign samples showed expression of VEGF_xxx_b. All but one sample also showed VEGF_165_ expression. (**B**) RT–PCR of RNA extracted from malignant prostate chips. Only four of the nine malignant samples showed expression of VEGF_165_b and eight out of nine malignant samples showed VEGF_165_ expression. *β*-microglobulin expression was seen in all the malignant tissues (data not shown). (**C**–**F**) Images of tumour-bearing mice. Tumours from PC3 cells overexpressing empty pcDNA_3_ vector (**C**), VEGF_165_ (**D**), VEGF_165_b (**E**) and cotransfection with VEGF_165_ and VEGF_165_b (**F**). Scale bar=10 mm. (**G**) VEGF_165_b reduced prostate tumour growth in a xenograft mouse model. Three million PC3 cells were injected and VEGF_165_b overexpression reduced control and VEGF_165_-mediated tumour growth at day 18 (*P*<0.05 Kruskal–Wallis, ^*^control *vs* VEGF_165_b *P*<0.05, ^+^VEGF_165_ *vs* VEGF_165_b *P*<0.05).

RNA was extracted from human prostate chips from patients undergoing TURP and expression of VEGF_165_ and VEGF_165_b mRNA was analysed by isoform specific PCR. Expression of VEGF_165_b was found in 10 out of 11 benign samples and VEGF_165_ was also present in 10 out of 11 (see Figure 1A). In malignant prostate on the other hand, VEGF_165_b was only found in four out of nine, whereas the VEGF_165_ expression was found in eight out of nine (see Figure 1B).

Human prostate cancer cells, PC3 cells, were transfected with control, VEGF_165_b, VEGF_165_ expression plasmids, or a combination of the two and injected into nude mice. The tumour volume was monitored over time and at day 18 the tumour volumes were significantly different (*P*<0.05). Figure 1C–F illustrates representative images of each group. The pro-angiogenic VEGF_165_ transfected cells produced rapidly growing tumours although not statistically different from control tumours (*P*>0.5). Conversely the VEGF_165_b transfectants resulted in smaller tumours (*P*<0.05 at 22 days compared with pcDNA3 controls), and those expressing both isoforms were significantly smaller than those expressing VEGF_165_ only (*P*<0.05 at 18 days).

### VEGF_165_b overexpression reduces renal cell carcinoma growth

Renal carcinoma (CAKI) cells, transfected with the different VEGF isoforms were analysed for VEGF expression. VEGF_165_b was undetectable in empty vector and VEGF_165_-transfected cells and VEGF_165_b expression was readily detectable in the VEGF_165_b- or cotransfected cells (Table 1). The endogenous levels of VEGF_165_ were high in CAKI cells and transfection of VEGF_165_ increased the total levels (Table 1).

**Figure 2:**
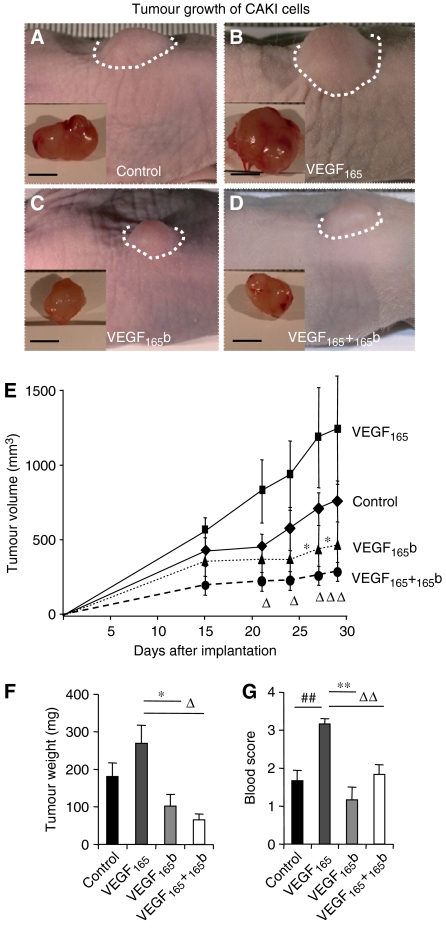
VEGF_165_b inhibits tumour growth in renal cell carcinomas. Renal cell carcinoma (8 × 10^6^ CAKI) cells, transfected with pcDNA_3_ vectors expressing empty vector (**A**) VEGF_165_ (**B**), VEGF_165_b (**C**) or cotransfected with VEGF_165_ and VEGF_165_b (**D**) were injected into the back of nude mice, *n*=6 mice per group, photographs taken at 29 days. Tumour border outlined by dotted line. Inset shows excised tumours. (**E**) Tumour growth curves. (**F**) Tumour weight at day of culling. (**G**) Macroscopic estimation of blood content at day of culling. (^*^P<0.05 VEGF_165_b *vs* VEGF_165_, ^Δ^*P*<0.05 ^ΔΔΔ^*P*<0.001 VEGF_165_+_165_b *vs* VEGF_165_, ^##^*P*<0.01 VEGF_165_ *vs* pcDNA).

Eight million transfected CAKI cells were injected into nude mice and monitored over time. Injection of control transfected cells resulted in solid, bloody tumours after 29 days (see Figure 2A), and VEGF_165_-expressing tumours resulted in larger, bloody tumours after 29 days (see Figure 2B), VEGF_165_b-expressing tumours, while they did grow (see Figure 2C) were smaller, and less bloody. Cells expressing both isoforms also resulted in small, relatively blood-free tumours that were not different from the VEGF_165_b-expressing tumours (see Figure 2D). Figure 2E shows that VEGF_165_b-expressing tumours grew significantly slower than VEGF_165_-expressing tumours (overall *P*<0.001, VEGF_165_ *vs* VEGF_165_b *P*<0.05 at day 27, VEGF_165_ *vs* both *P*<0.05 day 24 and *P*<0.001 day 27). No significant difference was found between tumours formed from cells expressing VEGF_165_ and control or between those expressing VEGF_165_b and both VEGF isoforms.

Upon excision of the tumours, they were weighed and scored blindly for blood content. VEGF_165_-expressing tumours were significantly larger (see Figure 2F) than control and VEGF_165_b-expressing tumours, which also resulted in a significant reduction of tumour volume (*P*<0.05 VEGF_165_ *vs* both isoforms or VEGF_165_b). There was a non-significant reduction in tumour volume for VEGF_165_b-expressing tumours compared with control transfected. Macroscopically the tumours differed with more blood in VEGF_165_-expressing tumours compared to any other of the groups (*P*<0.01, VEGF_165_ *vs* either of the other groups, Figure 2G). Similar data was observed with a smaller initial tumour cell injection (2 × 10^6^ cells, data not shown).

**Figure 3:**
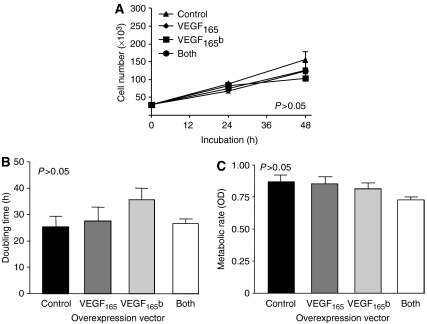
Overexpression of VEGF_165_b does not affect proliferation when overexpressed in renal cell carcinoma cells *in vitro*. (**A**, **B**) Transfected renal cell carcinoma CAKI cell growth was analysed by direct counting of cells after 24 and 48 h in low serum (0.01% FCS). The doubling time was calculated for each cell population. No significant differences were observed in either instance (*P*>0.05 *n*=3, one-way ANOVA). (**C**) Cell viability and metabolic rate was analysed at 48 h in low serum with transfected CAKI cells. No significant difference was observed (*P*>0.05 *n*=4, one-way ANOVA).

The growth rates of the transfected CAKI cells were analysed. There was no difference in the proliferation as measured by direct counting of cells (see Figure 3A and B) or analysis of metabolic rate (see Figure 3C). Both experiments were performed in the presence of serum with the same results (data not shown).

These results indicate that VEGF_165_b overexpression reduced tumour growth in renal cell carcinoma cells grown in mice even in the presence of VEGF_165_. The tumour inhibition by VEGF_165_b appears not to be through reduction in tumour cell proliferation.

### VEGF_165_b overexpression reduced Ewing's sarcoma growth and tumour-conditioned media reduced endothelial cell proliferation and migration

**Figure 4:**
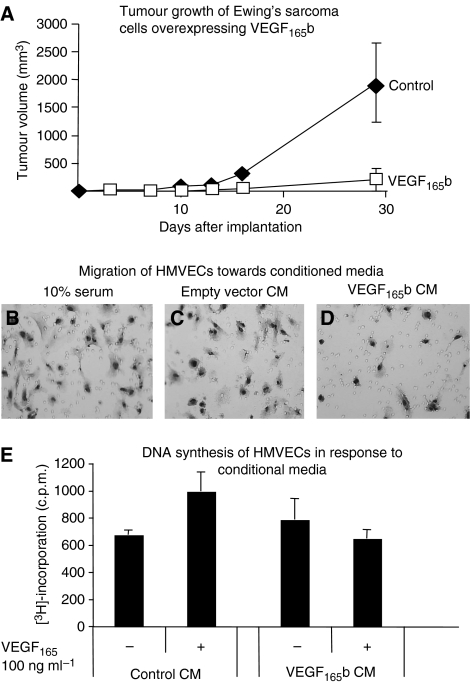
VEGF_165_b transfection reduces migration, proliferation and tumour growth *in vivo* of Ewing's sarcoma tumours. (**A**) VEGF_165_b overexpression in Ewing's sarcoma cells resulted in significantly smaller tumours 30 days after implantation of 1 × 10^6^ cells, *P*<0.05 after 7 days, one way ANOVA. (**B**) Human microvascular endothelial cells, HMVECs (stained with haematoxylin), migrated towards 10% serum and to conditioned media from Ewing's sarcoma cells (**C**). In contrast VEGF_165_b overexpression by these cells reduced migration compared to conditioned media and 10% serum (**D**). (**E**) When HMVECs were incubated in conditioned media from tumour cells VEGF_165_ (100 ng ml^−1^) could still stimulate increased proliferation. Conditioned media from cells overexpressing VEGF_165_b inhibited this increase.

Injection of TC71 Ewing's sarcoma cells overexpressing VEGF_165_b subcutaneously into the back of nude mice resulted in tumours that grew significantly slower than control cells over a time period of 29 days (see Figure 4A). To determine whether VEGF_165_b secreted from the tumour cells was active on the cells that go on to form blood vessels, conditioned media from the Ewing's sarcoma cells, TC71 cells, overexpressing VEGF_165_b was used to study migration of human microvascular vein endothelial cells, HMVEC. Fetal calf serum resulted in migration of HMVEC as expected (see Figure 4B), and conditioned media from control-transfected tumour cells induced a similar level of migration of HMVEC (see Figure 4C). VEGF_165_b overexpressing tumour cells resulted in less migration compared to tumour-conditioned media or control-stimulated cells (see Figure 4D).

To determine whether VEGF_165_b could inhibit growth of endothelial cells, DNA synthesis in HMVEC was measured. VEGF_165_ increased [^3^H]-incorporation into HMVECs when added to control tumour cell-conditioned medium. VEGF_165_b-conditioned media did not increase DNA synthesis of HMVEC cells and reduced VEGF_165_-mediated DNA synthesis (see Figure 4E). However, VEGF_165_b did not reduce proliferation below the level of control-conditioned media, indicating that the inhibition of proliferation was specific for VEGF_165_-mediated proliferation.

### Rate of tumour growth depends upon the VEGF isoform expression

**Figure 5:**
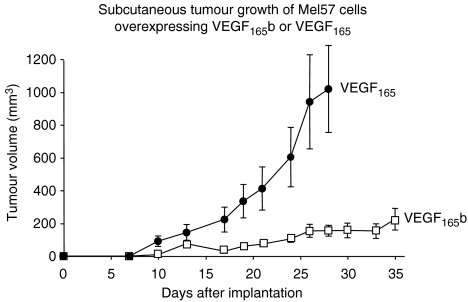
Switching expression from VEGF_165_ to VEGF_165_b inhibits tumour growth. Mel57 melanoma cells, which express very low levels of VEGF *in vivo* were transfected with VEGF_165_ or VEGF_165_b and 1 × 10^6^ cells injected subcutaneously into nude mice. Whereas the VEGF_165_ transfected cells grew rapidly, VEGF_165_b transfected cells grew no more quickly than previous studies have shown for this VEGF-deficient cell type.

The results above suggest that switching expression of VEGF from VEGF_165_ to VEGF_165_b might result in reduced tumour growth rates. To determine whether this was the case a VEGF-deficient melanoma cell line (Mel57), which normally grows slowly by co-option of existing vasculature (Westphal *et al*, 2000) was transfected with VEGF_165_ or VEGF_165_b, and implanted subcutaneously. Figure 5 shows that whereas VEGF_165_-expressing tumours rapidly grew, VEGF_165_b expressing-tumours were very slow growing, matching that of the previously published co-option dependent parental cell line, indicating that switching expression from VEGF_165_ to VEGF_165_b by altering splicing may be a useful therapeutic strategy.

### VEGF_165_b inhibits VEGF_165_-mediated tumour vessel in-growth

**Figure 6:**
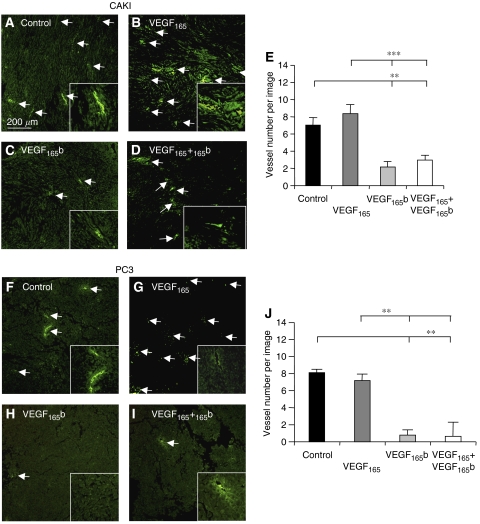
VEGF_165_b reduces vessel density in tumours. Tumour sections were stained with PECAM antibody to visualise vessels. (**A**–**D** and **F**–**I**) Representative images at × 10 magnification and × 20 (image inset). Quantification of vessel number in CAKI tumours (**E**) and PC3 (**J**). Overall *P*<0.0001 One-way ANOVA *P*<0.0001 ^**^P<0.01, ^***^P<0.001 compared to control or VEGF_165_.

To examine the mechanism for the reduction of tumour growth *in vivo* and the proposed anti-angiogenic effect of VEGF_165_b, tumour vessels were visualised by PECAM staining of excised tumour sections. In the CAKI tumours there was a significant increase in microvascular density from 7.03±0.86 per high power field in control (Figure 6A) to 8.37±1.06 in VEGF_165_-expressing tumours (Figure 6B). In contrast, there was a significant reduction in MVD to 2.17±0.65/hpf in VEGF_165_b-expressing tumours (*P*<0.01 compared with control Figure 6C), indicating that VEGF_165_b inhibited vessel growth in CAKI tumours. This was also significantly lower than VEGF_165_ (*P*<0.001). Furthermore, tumours in which VEGF_165_b and VEGF_165_ were coexpressed also had significantly reduced MVD (2.98±0.56, Figure 6D, *P*<0.001 compared with VEGF_165_), indicating that VEGF_165_b inhibited VEGF_165_-mediated blood vessel growth (Figure 6E). Similar results were seen in PC3 cells (Control 8.13±0.40, VEGF_165_ 7.17±1.66, VEGF_165_b 0.78±0.78, VEGF_165_+VEGF_165_b 0.64±0.64. see Figure 6F–J).

## DISCUSSION

VEGF has been generally considered in over 20 000 papers since 1990 as a pro-angiogenic tumour-enhancing endothelial-specific growth factor (Ferrara, 2002) and successful antiangiogenic agents have been directed at VEGF in cancer and eye disease (Gragoudas *et al*, 2004; Hurwitz *et al*, 2004). In 2002, we identified for the first time that an alternative splice site in the terminal exon 8 of the VEGF mRNA could be used to generate an alternative isoform (Bates *et al*, 2002), VEGF_165_b, which we subsequently showed to be one of a family of VEGF isoforms generated by C-terminal distal splice site selection, the VEGF_xxx_b family of isoforms (Perrin *et al*, 2005). However, while the conventional exon 8a containing isoforms predominate in the pathological angiogenic phenotype seen in tumours, proliferative retinopathy, arthritis etc (Ferrara, 2002), the exon 8b containing isoforms, which are anti-angiogenic *in vivo* are downregulated in a number of pathologies (Bates *et al*, 2002; Woolard *et al*, 2004; Cebe Suarez *et al*, 2006; Schumacher *et al*, 2007). Loss of the C-terminal domain (resulting in VEGF_159_) results in a loss of angiogenic activity of the VEGF molecule, but does not result in inhibition of angiogenesis (Cebe Suarez *et al*, 2006). The mechanism of action through which VEGF_xxx_b prevents tumour growth is not yet fully elucidated. However, it is clear from previous studies that VEGF_165_b is able to bind both VEGFR-1 (Cebe Suarez *et al*, 2006) and VEGFR-2 (Woolard *et al*, 2004), but initiates only weak signalling of the receptor to induce tyrosine phosphorylation (Woolard *et al*, 2004; Cebe Suarez *et al*, 2006), and is unable to induce a behavioural change in large vessel endothelial cells that mimic that shown by microvascular endothelial cells *in vivo* during angiogenesis (Woolard *et al*, 2004; Cebe Suarez *et al*, 2006). This study indicates for the first time that VEGF_165_b can exert its action by preventing tumour secreted endothelial growth factors (presumably VEGF_165_, or other VEGF_xxx_ isoforms) from acting on microvascular endothelial cells.

Several studies have shown that VEGF expression in the malignant tissue and/or plasma correlates with aggressive disease (reviewed in Delongchamps *et al*, 2006) and VEGF mRNA and protein are upregulated in prostate carcinoma (Ferrer *et al*, 1997; Jackson *et al*, 1997). Our data indicate that there is a switch in VEGF expression allowing the pro-angiogenic VEGF_xxx_ isoforms to dominate within malignant prostate and renal cell carcinoma, allowing the tumours to develop their own blood supply.

The current findings indicate that VEGF_165_b may have a therapeutic role in cancer treatment, by altering splicing of the VEGF gene to result in over-expression of VEGF_165_b at the expense of VEGF_165_. The latter mechanisms (control of splicing at the C-terminal end of the VEGF gene), is therefore one of intense interest, but unfortunately almost nothing has been published concerning the regulation of splicing of the VEGF gene. The vascular phenotype in both pathological and physiological angiogenesis may therefore depend on the balance of VEGF isoforms. We have speculated then that in addition to malignant change related to cell turnover/survival (which may also be determined by splicing (Venables, 2004) a second event occurs in which splicing control of many factors with pre- and antiangiogenic splice variants occur to allow the malignant disease to progress (Ladomery *et al*, 2006).

To conclude, these findings indicate that VEGF_165_b is able to inhibit growth of at least three different tumour types, and that the mechanism of inhibition is through inhibiting angiogenesis rather than a direct effect on tumour cell growth.

## Figures and Tables

**Table 1:** VEGF expression in transfected CAKI cells

**Transfection**	**VEGF_165_b (pg ml^−1^)**	**Total VEGF (pg ml^−1^)**
pcDNA	—	906
VEGF_165_	—	1020
VEGF_165_b	1562	1797
VEGF_165_+_165_b	975	1424

ELISA performed on conditioned media after 48 h from 200 000 transfected and selected CAKI cells.
